# Bumblebee Behavior on Flowers, but Not Initial Attraction, Is Altered by Short-Term Drought Stress

**DOI:** 10.3389/fpls.2020.564802

**Published:** 2021-01-13

**Authors:** Rebecca J. Höfer, Manfred Ayasse, Jonas Kuppler

**Affiliations:** Department of Biology, Institute of Evolutionary Ecology and Conservation Genomics, Ulm University, Ulm, Germany

**Keywords:** soil water availability, temperature, climate change, floral traits, pollinator behavior, Brassicaceae

## Abstract

Climate change is leading to increasing drought and higher temperatures, both of which reduce soil water levels and consequently water availability for plants. This reduction often induces physiological stress in plants, which in turn can affect floral development and production inducing phenotypic alterations in flowers. Because flower visitors notice and respond to small differences in floral phenotypes, changes in trait expression can alter trait-mediated flower visitor behavior. Temperature is also known to affect floral scent emission and foraging behavior and, therefore, might modulate trait-mediated flower visitor behavior. However, the link between changes in flower visitor behavior and floral traits in the context of increasing drought and temperature is still not fully understood. In a wind-tunnel experiment, we tested the behavior of 66 *Bombus terrestris* individuals in response to watered and drought-stressed *Sinapis arvensis* plants and determined whether these responses were modulated by air temperature. Further, we explored whether floral traits and drought treatment were correlated with bumblebee behavior. The initial attractiveness of drought and watered plants did not differ, as the time to first visit was similar. However, bumblebees visited watered plants more often, their visitation rate to flowers was higher on watered plants, and bumblebees stayed for longer, indicating that watered plants were more attractive for foraging. Bumblebee behavior differed between floral trait expressions, mostly independently of treatment, with larger inflorescences and flowers leading to a decrease in the time until the first flower visit and an increase in the number of visits and the flower visitation rate. Temperature modulated bumblebee activity, which was highest at 25°C; the interaction of drought/water treatment and temperature led to higher visitation rate on watered plants at 20°C, possibly as a result of higher nectar production. Thus, bumblebee behavior is influenced by the watered status of plants, and bumblebees can recognize differences in intraspecific phenotypes involving morphological traits and scent emission, despite overall morphological traits and scent emission not being clearly separated between treatments. Our results indicate that plants are able to buffer floral trait expressions against short-term drought events, potentially to maintain pollinator attraction.

## Introduction

With changing climate, drought periods and temperatures will increase (Intergovernmental Panel on Climate Change [IPCC], 2014), leading to reduced soil water levels, and might lead to physiological stress in plants (Beier et al., 2012). Abiotic stress is well-known to induce phenotypic changes in vegetative traits (Cornwell and Ackerly, 2009; Jung et al., 2014; La Rosa et al., 2019) but can also affect floral development and production, resulting in phenotypic alterations in flowers (Galen, 2000; Strauss and Witthall, 2006; Descamps et al., 2018). Such changes in floral trait expression can alter trait-mediated flower-visitor interactions and behavior, as flower visitors are able to notice and respond to small intraspecific differences in floral phenotypes within one species (Thomson et al., 1982; Conner and Rush, 1996; Mothershead and Marquis, 2000; Kuppler et al., 2016). However, the linkage between intraspecific responses to abiotic stress and different behaviors, e.g., number of visited flowers, remain unclear.

Drought is a complex term that can be defined in various ways, e.g., meteorological drought can be defined as “the number of days with precipitation less than some specified threshold” or to reduced groundwater or reservoir levels as characteristics of agricultural drought (National Drought Mitigation Center [NDMC], 2020). Generally speaking, drought is considered as a time span of unusual dry weather long enough to cause a severe hydrological imbalance (Intergovernmental Panel on Climate Change [IPCC], 2014) that depends on the amount, intensity, and timing of precipitation (National Drought Mitigation Center [NDMC], 2020) and the relationship of these parameters to historical data (Slette et al., 2019). Here, we use and define drought in the broad sense of “prolonged absence or marked deficiency of precipitation” (Slette et al., 2019) and as a synonym for reduced water availability. Short- and long-term drought periods are employed here to describe time spans that can last from several days or weeks for short-term to many months or even years for long-term drought.

Drought is known to cause intraspecific changes in trait expression (Jung et al., 2014) that can increase the stability of communities against environmental changes (Oney et al., 2013; Kuppler et al., 2016; Moran et al., 2016). Drought responses are known to modulate vegetative plant traits, e.g., reducing specific leaf area (Quiroga et al., 2013), to increase water-use efficiency and reduce water loss and prevent dehydration (Ludlow, 1989; Kooyers, 2015). Such responses are not limited to vegetative traits but can also be present in floral traits. For example, a decrease in flower size, in the number of flowers and/or in floral height under drought conditions can reduce transpirational water loss through flowers (Feild et al., 2009; Teixido and Valladares, 2014; Lambrecht et al., 2017) and can decrease water consumption for flower maintenance (Galen et al., 1999). However, these changes are often species-specific (Burkle and Runyon, 2016; Descamps et al., 2018, 2020; Glenny et al., 2018).

Phenotypic changes in floral traits often exert an influence on floral visitors, e.g., a reduced corolla length or reduced floral size leads to a reduced visitation rate (Burkle and Runyon, 2016; Gallagher and Campbell, 2017). So far, most studies have focused on observed visitation rates. However, the connection between visitation rate and pollination is not always straight forward but is dependent on pollinator behavior on plants and flowers (Engel and Irwin, 2003; Ne’eman et al., 2010). For example, difference*s* in visit durations or number of flowers per plant visited can affect the amount of pollen deposited (Ohara and Higashi, 1994; King et al., 2013) and the amount of pollen removal (Sahli and Conner, 2007). Therefore, an understanding of the possible changes brought about in pollinator behavior, e.g., flower visit duration, by drought stress in plants should provide greater insights into the effect of drought on pollination.

In addition to the modulation of morphological floral traits under drought conditions (Carroll et al., 2001; Caruso, 2006; Halpern et al., 2010; Burkle and Runyon, 2016; Glenny et al., 2018; Descamps et al., 2020; Walter, 2020), plasticity can also occur in scent emission. Drought stress increases floral scent emission and causes a shift in the composition of floral VOCs as some compound pathways might be up- or down-regulated. Studies showed an increase under drought for some compounds such as: (Z)-3-hexenol, 6-methyl-5-hepten-2-one, benzaldehyde, α- and β-pinene, and (E)-β-ocimene, (E,E)-α-farnesene (Burkle and Runyon, 2016; Glenny et al., 2018; Campbell et al., 2019; Rering et al., 2020).

Further, drought is usually associated with temperature increases, which can also affect floral scent. Under increasing temperature, floral scent emission can increase within minutes (Farré-Armengol et al., 2014), and a positive effect of warming on scent emission is generally assumed (Jamieson et al., 2017). However, temperature can also affect the relative contribution of the various compounds within the scent bouquet, e.g., an increase in aromatic hydrocarbons (Jamieson et al., 2017) and terpenes (Farré-Armengol et al., 2014). In combination with the direct effect of temperature on pollinator activity (Kühsel and Blüthgen, 2015), these changes are likely to alter the visitation patterns and behavior of pollinators that rely on such information for the detection of suitable flowers (Junker and Parachnowitsch, 2015). Therefore, temperature and drought can independently act upon floral traits, flower-visitor interactions, and pollinator behavior but may also have interacting effects (Jamieson et al., 2017). An exploration of their combined effects should elucidate these linkages.

In this study, we have examined (1) the effects of drought on floral morphology, phenology, and scent emission*;* (2) the way in which such phenotypic changes influence bumblebee behavior; and (3) possible interactions between temperature and drought on floral traits and trait-mediated bumblebee behavior. Bumblebee – or other flower visitor – behavior in response to plants and their phenotypic expression under various conditions can conveniently be observed in a wind tunnel. First, in a climate-controlled room ambient conditions such as airflow, humidity and temperature can be readily controlled in this set-up, and second, due to a constant airflow, flower visitor behavior in response to scent plums can be explored (Dötterl et al., 2006; Klahre et al., 2011). We have tested in a wind tunnel whether a combination of short-term drought stress and changes in temperature result in altered pollinator behavior. Additionally, we have measured floral traits to determine whether altered bumblebee behavior is linked with possible phenotypic changes in floral traits induced by drought stress and altered temperatures.

## Materials and Methods

### Plants and Drought Treatment

*Sinapis arvensis* L. (wild mustard, Brassicaceae) is an annual, self-incompatible, cruciferous plant native to southern and middle Europe. It attracts a broad range of flower visitors, mostly bees, and syrphid flies (Kunin, 1993). *S. arvensis* and other Brassicaceae generally grow in meadows and agricultural landscapes and provide important resources for multiple pollinator species. Therefore, they can be seen as a representative of a common widespread plant with a generalist pollination system. We obtained seeds of *S. arvensis* from wild populations in southern Germany (purchased from Rieger-Hofmann GmbH, Blaufelden, Germany). The seeds were treated with aqueous gibberellic acid solution (1000 ppm; Sigma, St. Louis, MO, United States) and left on wet filter paper in darkness at room temperature until germination. Subsequently the seeds were transferred into 0.6-liter-pots containing a soil mixture of 3:1 peat:sand. Once the cotyledons had emerged (∼3 days), we transplanted the seedlings individually into 0.6-liter pots containing a soil mixture of 3:2:1 TKS 2:compost:sand (TKS2, Floragard Vertriebs-GmbH, 26135 Oldenburg, Germany). We reared 20 plants per batch per week (in total, six batches). Plants were kept in a phytochamber (Phytotron 1, Vötsch Industrietechnik GmbH, Balingen, Germany) in the Botanical Garden of Ulm University at 20°C and 66% relative humidity with a 12:12 day:night cycle at a light intensity of 500 μmol m^–2^ s^–1^. Plants were randomly grouped into pairs consisting of a control and a drought-stressed plant (in total, 20 plant pairs were used). Control plants were watered daily once with 100 ml water. Drought-stressed plants were watered once every other day with the same amount of water. This pulsed drought treatment, which started 2–3 days before flowering and lasted for 18 days (Burkle and Runyon, 2016), resembles a short-term drought period similar to that prevailing in the field and has often been used in drought stress studies. We also tested longer periods of drought, but after 2 days without water, mortality, or signs of severe drought stress were observed. Soil humidity was controlled using a self-made soil humidity sensor with an Arduino system (Iduino ME110, Arduino software version 1.8.8, board: Genuino Uno). The applied drought stress significantly reduced the soil humidity of the drought-stressed plants [mean (SD)%: **watered:** 34.8 (5.2)%, **drought-stressed**: 24.7 (9.2)%; and Wilcoxon Rank-Sum test: *W* = 356180, *p* < 0.001].

### Bumblebees

As a common visitor of *S. arvensis*, we used *Bombus terrestris* L. (Apidae) from self-reared colonies at the Institute of Evolutionary Ecology and Conservation Genomics at Ulm University (Rottler et al., 2013; Rottler-Hoermann et al., 2016) for our behavioral assays. The founding queens were descendants of commercial colonies (Koppert Biological Systems, Netherlands). The colonies were kept in wooden boxes (39 cm × 16.5 cm × 16 cm) in constant darkness at a temperature of 27°C and a relative humidity of 60%. The bumblebees were provided *ad libitum* with a 55% sugar solution of API-Invert^®^ (Südzucker AG, Mannheim, Germany) and fresh pollen (Koppert Biological Systems, Netherlands).

### Flower-Visitor Interactions

For the behavioral tests, we used two-month-old colonies with about 30 workers (in total, six colonies were used). Bumblebee behavior on watered and drought-stressed plants was investigated by conducting a two-choice bioassay in a wind tunnel (200 cm × 80 cm × 80 cm). 2 days before the experiments, the colony was connected via a tube (length 30 cm, diameter 1.5 cm) to the wind tunnel to allow the bumblebees to acclimatize to and to forage within the wind tunnel. The bumblebees were provided *ad libitum* with the above sugar solution and fresh pollen within the wind tunnel. After 2 days, the colony was removed from the wind tunnel and connected to a flight cage via a tube (60 cm × 60 cm × 60 cm, BugDorm, MegaView Science Co., Ltd., Taiwan) with the same food provision. On the morning of the experiments, individual bumblebees were caught, marked individually, and starved until they were used.

A watered and a drought-stressed plant were placed next to each other at a distance of 30 cm in the middle of the wind tunnel. A fan (D440/E15 with an FDR 32 speed controller; Fischbach, Neunkirchen, Germany) blew charcoal-filtered air through the tunnel. A single bumblebee was placed in the tunnel at the end opposite to the fan. The bumblebee was allowed to acclimatize for at least 5 min. After this time, any bumblebees that did not start to fly or were otherwise active with regard to the plants were removed and excluded from the analysis (excluded individuals, *N* = 49). Bumblebees (*N* = 66) were observed for a maximum of 10 min (Hoover et al., 2012) with the following behavior types being recorded: (1) time to first visit [sec], (2) number of approaches (≤5 cm distance to flower), (3) number of landings, (4) number of all visits (sum of approaches and landings), (5) number of visited flowers per landing, (6) duration of landings [sec], (7) visitation rate, which was calculated as the number of visited flowers during a landing divided by the total flower number of the plant individual per min, and (8) relative duration, which was calculated by dividing duration with the total active time during the observation. Between the replicates, the wind tunnel was cleaned with unscented soap, and the position of plants and plant pairs were switched regularly.

### Temperature

To test whether air temperature affected bumblebee behavior, we performed the wind tunnel experiment at three different air temperatures. The day before the trial, the air temperature in the wind tunnel room was set to the relevant temperature: 20, 25, or 30°C. If possible, we observed the same bumblebees with their corresponding plant pairs at all three temperatures (bumblebees: **20°C** *N* = 35; **25°C** *N* = 33; **30°C** *N* = 37; and plant pairs: 12 pairs per temperature).

### Trait Measurements

To test differences in plant phenotype between treatments, we measured eight morphological floral traits and nectar volume, which are all known to mediate flower-visitor interactions (Thomson et al., 1982; Stang et al., 2006; Junker et al., 2013; Kuppler et al., 2016). Flower height (height of the highest flower) [cm], display size of flowers (greatest expansion of flower) [mm], display size of inflorescences (greatest expansion of inflorescences, either vertical or horizontal) [mm], number of flowers and inflorescences, calyx length [mm], style length [mm], and longest stamen (filament plus anther) [mm] were measured directly on the plant by using a digital caliper (Traceable^®^ Digital Caliper 6-inches, VWR International LLC, Leuven, Belgium), except for flower height (measured with a folding yardstick). Nectar volume per flower [μl] was measured via a glass capillary (0.5 μl). All measurements were taken on three freshly open flowers or inflorescences from a low, middle, and high position to avoid position and age effects; means were used for statistical analyses. Trait measurements took place in the morning on the same day that we observed bumblebee behavior.

#### Scent Collection and Analysis

We used dynamic headspace to examine the effect of drought and temperature on the quality and quantity of scent emission. All inflorescences of each plant were enclosed within an oven bag (Toppits^®^, Minden, Germany) from which the air was pumped for 10 min to remove ambient air. After 20 min of scent enrichment, the emitted volatiles were trapped for 3 min on 1.5 mg Tenax (mesh 60–80; Supelco, Bellefonte, PA, United States) and 1.5 mg Carbotrap B (mesh 20–40; Supelco) in a quartz vial (length 20 mm, inner diameter 2 mm) by using a membrane pump (G12/01 EB; ASF Rietschle-Thomas, Puchheim, Germany) with a flow rate of 200 ml min^–1^. Scent enrichment and trapping were repeated; thus, the total sample time was 46 min for each plant. All samples were collected between 08:00 and 12:00 h. The temperature in the room with the wind tunnel was controlled by a thermostat. Plant pairs spent at least 15 h at the respective temperature before scent collection. Scent samples were analyzed using an automatic thermal desorption system (TDU, Gerstel, Mühlheim a. d. Ruhr, Germany) and a cold-injection apparatus (CIS 4C, Gerstel) coupled with a GC-MS (7890B GC–5977A MSD, Agilent Technologies, Germany). The GC-MS was equipped with a DB-5MS silica column (5% diphenyl, 95% dimethyl polysiloxane; 30 m long, inner diameter 0.25 mm, film thickness 0.25 μm), and the column flow (carrier gas: helium) was set to 1.5 ml min^–1^. The GC oven temperature was initially at 40°C, was then increased by 6°C per minute to 250°C, and subsequently held constant for 1 min. The MS interface was set at 250°C. Mass spectra were taken at 70 eV (in EI mode) from m/z 30 to 350. The GC/MS data were analyzed using the GCMSolution package (Version 2.72, Shimadzu Corporation, Kyoto, Japan). Compounds were identified using the mass spectral libraries Wiley 9, Nist 2011, FFNSC 2, and Adams, 2007. The compounds found in flowers were compared with those found in the blanks (empty oven bags) to determine which compounds were emitted in particular by flowers. The amount of each compound emitted was standardized by the number of flowers. Compounds were considered as being most common when they appeared in more than four plants per treatment.

### Data Analysis

We tested the effects of plant drought stress on bumblebee behavior. For all models, we used treatment and temperature as fixed factors and each bee nested in nest ID as random factors to account for differences between individuals and colonies. The effect of drought stress and temperature on time until the first visit, visitation rate, and total number of visits was analyzed using the *lmer*-function with restricted maximum likelihood (REML). The visitation rate was log10(*x* + 0.001)-transformed to achieve normal distribution. If no model convergence was reached after the default 10,000 iterations, we restarted the model from the previous fit with a maximum of 100,000 iterations. To investigate the effect of treatment and temperature on the relative duration, we used the *glmmTMB*-function from the *glmmTMB*-package (Brooks et al., 2017) with beta-family distribution. Therefore, the relative duration was transformed as suggested by Brooks et al., 2017: $\frac{\mathrm{dependent}\,\mathrm{variable}\times \left(n-1\right)+0.5}{n}$ with *n* being the sample size of the dependent variable.

Further, in order to test the association between bumblebee behavior and floral phenotype in dependence on treatment, we correlated bumblebee behavior with floral traits and drought stress with trait and treatment as fixed factors by using the same models as above. Time to first visit, the number of visits and visitation rate as the dependent variables were log-transformed or log10(*x* + 1)-transformed. We also tested the effect of floral scent emission on time until the first visit, number of visits, visitation rate, or relative duration as dependent variables and each bee nested in nest ID as random factors. All model fits were validated using the *DHARMa*-package (Hartig, 2020) and were adequate. Number of visits depending on flower height and number of inflorescences, and the visitation rate depending on flower height, the number of inflorescences and the number of flowers as a model fit showed an imperfect fit of error distribution. All data were analyzed and plotted using R (version 3.5.0, R Core Team, 2018), except for MDS of floral scent bouquet, which was analyzed and plotted with PRIMER-E (version 6.1.15, with PERMANOVA+, version 1.0.5; PRIMER-E Ltd., 2012).

In order to test whether differences in floral traits between drought-stressed and watered plants occurred, we performed linear mixed-effect models (LMMs) by using the *lmer*-function from the *lme4*-package (Bates et al., 2015) with treatment as the fixed factor, and the mean of flower height, number or size of inflorescences, number or size of flowers, calyx, style or stamen length, nectar volume, or total floral scent emission as dependent variables and the plant as the random factor by using REML. To achieve normal distribution, scent emission variable was log10 + 1-transformed. We used the Kruskal*–*Wallis test to analyze the effect of drought stress on the amount of emitted scent compounds per flower. The effect of drought treatment and temperature on floral scent bouquet was assessed by permutational multivariate ANOVA (PERMANOVA, 9999 permutations, Bray–Curtis similarity distance matrix). One sample from a plant in the drought treatment was empty and was therefore excluded.

## Results

### Bumblebee Behavior and Drought Stress

Initial attractiveness (= time to the first visit) did not differ between the two treatments (LMM: χ^2^ = 0.94, *p* = 0.332). However, the behavior of the bumblebees after the initial visit differed between treatments. Bumblebees visited watered plants more often than drought-stressed plants [mean (SD): **watered:** 10.9 (4.8); **drought-stressed**: 9.1 (4.5); and LMM: χ^2^ = 7.72, *p* < 0.01] and visited twice as much flowers per min (= visitation rate) of watered plants compared with drought-stressed plants [mean (SD) flowers/min: **watered:** 0.09 (0.12); **drought-stressed**: 0.04 (0.08); and LMM: χ^2^ = 9.12, *p* < 0.01]. Moreover, bumblebees spent on average more time on watered plants [mean (SD), **watered:** 0.7 (0.3); **drought-stressed**: 0.6 (0.4); and GLMM: χ^2^ = 4.25, *p* < 0.05].

### Effects of Drought Stress and Temperature on Behavior

**FIGURE 1:**
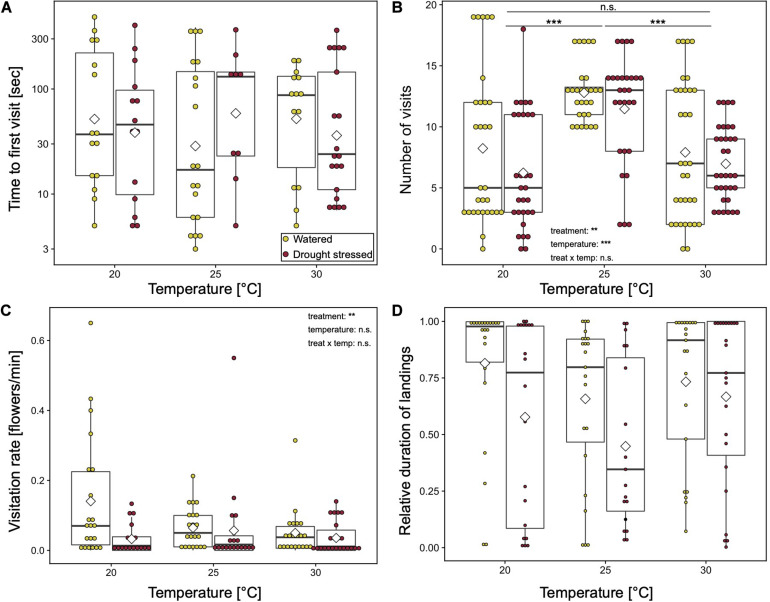
Behavior of bumblebees at various temperatures with respect to watered and drought-stressed plants. **(A)** Time to first plant visit [sec] (approach or landing); **(B)** number of visits per plant (approaches plus landings); **(C)** flower visitation rate per landing [min]; and **(D)** relative duration of landings. Boxplots show median range, interquartile range, and minimum/maximum range. White diamonds show mean value. Means of each bumblebee were compared using general linear mixed effect models. ***p < 0.001; **p < 0.01; and n.s., non-significant.

Temperature had no effect on time to first visit (LMM: χ^2^ = 1, *p* = 0.607, Figure 1A). Number of all visits per plant were significantly affected by temperature (LMM: χ^2^ = 29.37, *p* < 0.0001, Figure 1B). At 25°C, the number of visits was one and a half times higher than at the other temperatures [mean (SD): **20°C**: 8.7 (5.6); **25°C**: 13 (2.7); **30°C**: 8.5 (4.3); *Post hoc* Tukey-test: **20–25°C** *p* < 0.001, **20–30°C** *p* = 0.73, **25–30°C** *p* < 0.0001]. The visitation rate [mean (SD) flowers/min: **20°C**: 0.09 (0.15), **25°C**: 0.06 (0.1), **30°C**: 0.04 (0.06), LMM: χ^2^ = 0.6, *p* = 0.741] and the duration of landings [mean (SD): **20°C**: 0.68 (0.39), **25°C**: 0.58 (0.36), **30°C**: 0.7 (0.35), LMM: χ^2^ = 3.26, *p* = 0.596] showed a tendency to be higher at 20°C. The interaction of treatment and temperature had no significant effects on the behavior of the bumblebees. However, visitation rate and relative duration tend to be highest at 20°C on watered plants (Figures 1C,D).

Additionally, the percentage of bumblebees that participated in the experiments were higher at 25°C and 30°C (82.5% and 66.1%, respectively). Only 60.3% of the bumblebees participated at 20°C. However, this difference was not significant (GLM with binomial error: χ^2^ = 0.46, *p* = 0.499).

### Effects of Floral Traits and Drought Stress on Behavior

**FIGURE 2:**
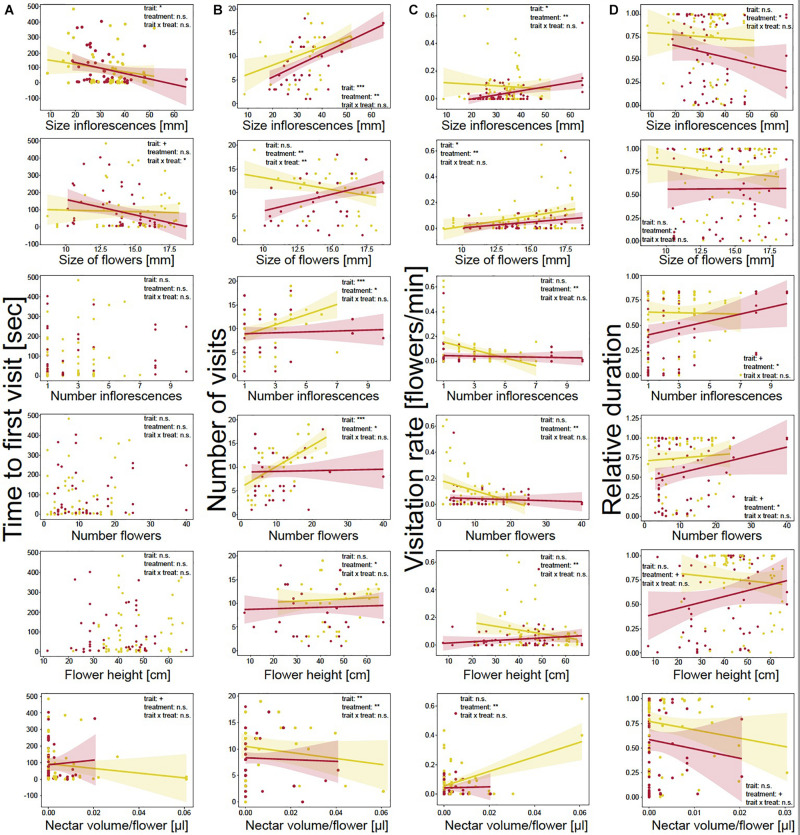
Behavior of bumblebees in correlation with measured morphological traits and drought treatment. **(A)** Time to first plant visit [sec] (approach or landing); **(B)** number of plant visits (approaches plus landings); **(C)** visitation rate of flowers [min]; and **(D)** relative duration of landings. Plant trait values are mean values. Each dot represents one event on a plant. Colored lines show trend lines for significant correlations of behavior and plant trait; colored areas show confidence interval. Correlations were measured using linear mixed-effect models. Significance levels are given as asterisks: ***p < 0.001; **p < 0.01;*p < 0.05; ^+^*p* < 0.10; and n.s., non-significant.

In addition, we tested whether floral traits and drought treatment correlated with time to first visit, number of visits per plant, visitation rate of flowers, and relative duration of landings (Figures 2A–D).

**FIGURE 3:**
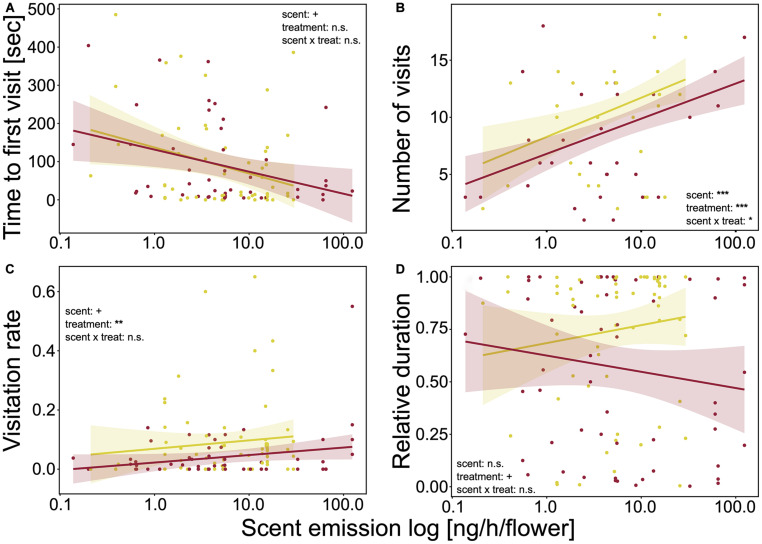
Behaviors of bumblebees in correlation with total scent emission [ng/h/flower] (logarithmic scale). **(A)** Time to first plant visit [sec] (approach or landing); **(B)** number of visits (approaches plus landings); **(C)** flower visitation rate per landing [min]; and **(D)** duration of landings, relative to active time of bumblebees. Colored lines show trend lines for significant correlations of behavior and plant trait; colored areas show confidence interval. Correlations were measured using linear mixed-effect models. Significance levels are given as asterisks: ***p < 0.001; **p < 0.01;*p < 0.05; ^+^*p* < 0.10; and n.s., non-significant.

*The time to first visit* was not influenced by treatment (Figure 2A and Supplementary Table 3), but was significantly affected by floral traits. Bumblebees needed less time until the first visit when plants had larger inflorescences (LMM: χ^2^ = 4.44, *p* = 0.035) and flowers (LMM: χ^2^ = 3.39, *p* = 0.066) and more nectar per flower (LMM: χ^2^ = 2.74, *p* = 0.098). Further, the decreases in time to first visit with increasing floral size was stronger for drought-stressed plants (LMM: χ^2^ = 4.61, *p* = 0.032). Increasing scent emission significantly decreased the time to first visit (LMM: χ^2^ = 3.20, *p* = 0.073; Figure 3A). For the other floral traits, neither trait, *nor* treatment, nor the interaction of the two factors had an effect on time to the first visit.

*Number of visits per plant* was also affected by treatment as bumblebees visited watered plants more often than drought-stressed plants for all correlated floral traits (Figure 2B and Supplementary Table 3). Measured floral traits also had significant effects on visits. The visits significantly increased for plants with larger inflorescences (LMM: χ^2^ = 23.23, *p* < 0.001) and more inflorescences (LMM: χ^2^ = 23.18, *p* < 0.001) and flowers (LMM: χ^2^ = 26.76, *p* < 0.001) and decreased with higher nectar volume per flower (LMM: χ^2^ = 7.54, *p* = 0.006). Flower size and height had no influence on the number of visits. However, the interaction of floral traits and treatment significantly influenced the number of visits as, with increasing floral size, the visits decreased for watered and increased for drought-stressed plants (LMM: χ^2^ = 7.63, *p* = 0.006). Higher scent emission (LMM: χ^2^ = 22.61, *p* < 0.001; Figure 3B) and the interaction of scent and treatment significantly affected the number of visits, as the visits increased more strongly for watered plants than for drought-stressed plants with increasing scent emission (LMM: χ^2^ = 6.01, *p* = 0.014, Figure 3B).

*Visitation rate* was significantly influenced by treatment, as the rate was higher on watered plants for all measured floral traits (Figure 2C and Supplementary Table 3). Furthermore, the rate was significantly affected by floral traits as it increased with increasing flower size (LMM: χ^2^ = 5.48, *p* = 0.019) and inflorescence size (LMM: χ^2^ = 6.08, *p* = 0.014). The other traits had no effect on visitation rate; however, a tendency was noted that visitation rate increased with nectar volume (Figure 2C and Supplementary Table 3). Scent emission positively affected the visitation rate (LMM: χ^2^ = 3.67, *p* = 0.055; Figure 3C).

*Relative duration of landings* was significantly affected by treatment, as bumblebees visited watered plants for longer periods (Figure 2D and Supplementary Table 3). However, duration was not significantly affected by floral traits, although tendencies were observed indicating that plants were visited for longer when they had more flowers (LMM: χ^2^ = 2.81, *p* = 0.094) or inflorescences (LMM: χ^2^ = 2.74, *p* = 0.098); landings were shorter with increasing nectar volume (LMM: χ^2^ = 1.25, *p* = 0.264). The interaction of floral trait and treatment had no effect on duration for all measured traits. Scent emission (LMM: χ^2^ = 0.1, *p* = 0.754, Figure 3D) and the interaction of scent and treatment had no effect on relative duration.

### Floral Traits and Drought Stress

**FIGURE 4:**
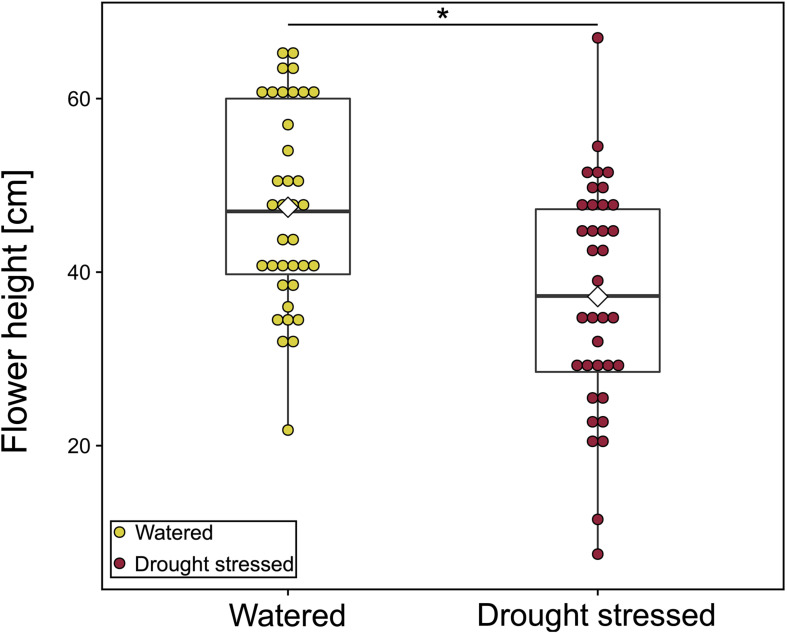
Flower height [cm] of watered and drought-stressed plants. Each colored dot shows mean value of one plant for all observation days. Watered: *N* = 36 [mean (SD) 47.5 (11.5) cm]; drought stressed: *N* = 36 [mean (SD) 37.2 (13.1) cm]. Boxplots show median range, interquartile range, and minimum/maximum range. White diamonds show mean height. Means of each plant were compared using linear mixed-effect models. *p < 0.05.

Drought-stressed plants were 10 cm smaller than watered plants [mean (SD) cm: **watered:** 47.5 (11.5) cm, **drought-stressed**: 37.2 (13.1) cm; LMM: χ^2^ = 4.03, *p* = 0.045, Figure 4 and Supplementary Table 1]. We found no significant effect of drought treatment for the other morphological traits, nectar volume and floral scent (Supplementary Table 1). Nevertheless, nectar volume per flower was twice as high in watered plants [mean (SD) μl: **watered:** 0.006 (0.012) μl, **drought**-**stressed:** 0.003 (0.005) μl; LMM: χ^2^ = 2.60, *p* = 0.107, Supplementary Table 1], and the highest amount was found in watered plants at 20°C. The flowers emitted the same 25 compounds in both treatments (Supplementary Table 2). Mean total scent emission per flower tended to be higher in drought-stressed plants (LMM: χ*^2^* = 0.06, *p* = 0.814; Supplementary Figure 1). Temperature (LMM: χ^2^ = 2.64, *p* = 0.267) and the interaction of treatment and temperature (LMM: χ*^2^* = 0.69, *p* = 0.707) had no significant effect on the total scent emission. The emission rate for single compounds did not differ between the two treatments (Supplementary Figure 2). The scent bouquet also did not differ between the two treatment groups (PERMANOVA: *Pseudo-F*_1_,_65_ = 0.81, *p* = 0.557; Supplementary Figure 3A). Temperature and the interaction of treatment and temperature also had no effect on the composition of scent bouquet (PERMANOVA: temperature*:* Pseudo*-F*_2_,_65_ = 1.06, *p* = 0.385; treatment × temperature*:* Pseudo*-F*_2_,_65_ = 1.07, *p* = 0.363; Supplementary Figure 3B). Additionally, we found that total scent emission was positively correlated with nectar amount per flower (LMM: χ^2^ = 5.01, *p* = 0.025).

## Discussion

Drought stress can alter a number of flower-visitor interactions, although little has been documented about the effects of drought stress on pollinator behavior, the way that this might be linked with induced phenotypic changes in flower morphology and floral scent emission, and the possible influences of a combination of temperature and drought on such behavior. Our results show that drought-stress treatment negatively affects bumblebee behavior. Drought-stressed plants are visited less frequent, and bumblebees stay for shorter periods on their flowers, but no difference has been found in the initial attraction, as measured by the time to the first visit. Bumblebees are more active under increased temperature, although we have not detected a clear interaction of treatment and temperature, despite the visitation rate tending to be highest on watered plants at 20°C. Further, we have shown that bumblebees respond to differences in plant phenotypes, being able to find those plants with larger inflorescences and/or higher scent emission much faster. However, variability in floral traits is generally large with drought-stressed plants growing smaller and tending to produce less nectar per flower. Overall, our study reveals that plants are at least partially able to compensate for induced drought stress by reducing plant growth in order to maintain reproductive traits for pollinator attraction. However, we have found trait-mediated differences in behavior between watered and drought-stressed plants, indicating that plants are not fully able to compensate for drought stress to maintain bumblebee behavior. Thus, if stress levels increase, they will have even greater impacts on plants and trait-mediated bumblebee behavior.

Whereas the initial attractiveness of plants in both treatments was similar for bumblebees, watered plants were visited more often than drought-stressed plants (see also Burkle and Runyon, 2016; Descamps et al., 2018). Additionally, the visitation rate of flowers and the number of visits per plant were higher on watered plants, and bumblebees remained for longer on these plants. As we have determined no differences in number of flowers or morphology between watered and drought-stressed plants, this behavior might be explained by the reduced nectar production per flower in the drought-stressed plants, as these plants are less attractive for foraging (e.g., Carroll et al., 2001; Waser and Price, 2016; Descamps et al., 2018). Indeed, nectar production is the highest in watered plants at 20°C, reflecting the pattern for the visitation rate. Bumblebees are able to optimize their foraging behavior by choosing flowers with higher nectar amounts thereby collecting more nectar in a shorter time (Chittka et al., 1997; Blarer et al., 2002; Cartar, 2004; Dreisig, 2012). This is the reason that the landing duration tends to decrease with higher nectar volume. Nectar production under normal circumstances is costly in terms of energy consumption (Southwick, 1984; Pyke, 1991). When plants are exposed to stressors such as drought or heat, resources may be not sufficient to compensate fully for the drought stress and for the maintenance of the reproductive organs and normal nectar production, leading to decreased nectar secretion in drought-stressed plants.

For successful pollination, not only pollinator attraction and visitation rate are important, but also the duration of visits. If bumblebees stay for shorter periods of time on each flower, pollen is less likely to be received by the stigma (Ohara and Higashi, 1994; King et al., 2013) or will be transferred from the anthers to the body of the pollinator (Sahli and Conner, 2007), potentially impacting female and male reproductive success. Further, the reduced number of flowers visited per plant suggest that not all flowers will be efficiently pollinated. In *Fagopyrum esculentum* (Polygonaceae), Rering et al. (2020) have shown that drought stress leads to reduced visits, decreased pollination success, and consequently lower seed set (see also Gallagher and Campbell, 2017). Therefore, increasing drought events and longer drought periods will influence bumblebee foraging behavior leading to fewer and shorter visits on drought-stressed plants and hence to reduced pollination success (Chagnon et al., 1989).

Bumblebee behavior differed between floral trait expressions independently of treatment in our experiments. Larger inflorescences and flowers decreased the time until the first visit and increased the number of visits and the flower visitation rate. This indicates that bumblebees are able to differentiate between the phenotypes of plants (Thomson et al., 1982; Conner and Rush, 1996; Hoffmeister and Junker, 2017). Additionally, with higher scent emission the number of visits increased. As the emission is positively correlated with nectar volume per flower, this may suggests that it is an honest signal for reward, namely that plants with a higher scent emission provide more food resources for bumblebees (Wright and Schiestl, 2009; Knauer and Schiestl, 2015). However, under drought stress, other plant species also emit more scent (Burkle and Runyon, 2016; Gallagher and Campbell, 2017) possibly to simulate the presence of nectar.

Temperature did neither affect the total amount of scent emission nor the composition of the scent compounds of watered and drought-stressed plant; this finding might be the reason that, under the two treatments, the flowers have a similar initial attractiveness to the bumblebees. Thus, *S. arvensis* is able to emit a stable scent bouquet even under drought stress and across various temperatures, potentially in order to maintain function in pollinator attraction. However, other studies have revealed that total scent emission increases because of higher vaporization up to a maximum of 30°C (Sagae et al., 2008; Scaven and Rafferty, 2013; Farré-Armengol et al., 2014), and that scent emission can change within 2 h (Hu et al., 2013). Such higher floral scent emissions attributable to increasing temperatures and drought (Burkle and Runyon, 2016) might also have negative effects. An increase of, for example, terpene emissions in floral parts and other tissues may involve higher metabolic costs by pathways producing these compounds (Farré-Armengol et al., 2014). Higher metabolic costs might then lower the plastic response of plants to drought stress. Furthermore, qualitative changes in floral scent bouquets brought about by drought stress and increasing temperatures (Llusià and Peñuelas, 2000; Farré-Armengol et al., 2014) possibly disturbs flower-visitor communication. The actual visitor species is no longer able to find its host plant (Vereecken et al., 2010), which in turn would lower visitation rate and pollination success.

In our study, we have shown that temperature in combination with drought stress in plants plays no significant role in bumblebee behavior. However, the pattern for visitation rate follows that of nectar production, with highest values occurring at 20°C for watered plants, indicating the interacting effects of drought and temperature on trait-mediated flower-bumblebee behavior.

Temperature on its own significantly influences bumblebee behavior, as the number of visits per plant was highest and most bumblebees were active and participating in the experiments at the medium temperature of 25°C. This corresponds to the reported temperature of the highest foraging activity at 25°C, whereas at 32.7°C foraging activity significantly decreased by 69.7%, indicating that 25°C is the optimal foraging temperature for *B. terrestris* and possibly supports their thermoregulation (Kwon and Saeed, 2003). Other studies have shown that, at lower ambient temperatures, bees prefer warmer flowers with warmer nectar to maintain body temperature (Dyer et al., 2006; Norgate et al., 2010; Shrestha et al., 2018). Similarly, at high temperatures bumblebees might adjust their behavior to avoid overheating by changing foraging patterns and floral preferences and handling. Thus, interacting effects between temperature and drought might especially occur under severe drought and temperature conditions.

*Sinapis arvensis* plants are able to grow and flower under our drought treatment. In congruence with other studies (Chaves et al., 2003; Burkle and Runyon, 2016; Kahl et al., 2019; La Rosa et al., 2019), our drought-stressed plants grow less than the daily watered control plants. However, we have found that flower size did not decrease as has commonly been observed (Carroll et al., 2001; Halpern et al., 2010; Burkle and Runyon, 2016) for plant species with similar moisture value to ours (Ellenberg et al., 1992). These differences might be explained by different growing conditions, because our plants were reared and kept in a phytochamber with constant temperature and light conditions. Under field or semi-natural conditions, such as in a greenhouse with fluctuating light and temperatures and the potential impact of herbivores or pathogens (Rusman et al., 2019), it may be more difficult for plants to compensate for water deficits. Herbivory mediates the effects of drought on floral size for certain plant species (Burkle and Runyon, 2016) and limits the plastic responses to herbivore damage during low water treatment (Halpern et al., 2010). However, several species have been shown to maintain floral trait expression under drought treatment (Caruso, 2006; Glenny et al., 2018; Walter, 2020). Thus, plants exposed only to one stressor may be more likely to compensate for drought stress by reducing growth to invest resources in floral parts for the maintenance of pollinator attraction based on visual information. Therefore, the determination of ranges of drought exposure in which plant species are still able to compensate for this stress in order to maintain their normal floral phenotype, in combination with other stressors, might represent an important step for predicting impacts of drought on the floral phenotype of plants.

Overall, our study has revealed that *S. arvensis* plants are able to maintain pollinator attraction under drought stress, but that bumblebee behavior changes during flower handling. Floral trait expression, largely independent of treatment, mediates bumblebee behavior. However, the response of bumblebees to certain floral trait expression, e.g., floral size and nectar amount, differs between drought and watered treatments. Thus, our results indicate that plants are able to buffer floral trait expressions against short-term drought, potentially maintaining the attractiveness of their flowers to ensure at least a few visits by pollinators. Nevertheless, we have found indications that the quality and quantity of pollinator visits are impaired by drought stress. Therefore, plants are able to withstand reduced water availability within a certain range. These findings highlight the need for a comprehensive understanding of the impacts of various drought intensities on plants for the planning of future drought management. Moreover, the impact of drought on possible changes of behavior of pollinators on flowers and the consequences for female and male reproductive success should be assessed in future studies.

## Data Availability Statement

The raw data supporting the conclusions of this article will be made available by the authors, without undue reservation.

## Author Contributions

JK conceived the study. RH, MA, and JK designed the study. RH collected the data. RH and JK analyzed the data. RH drafted the first version of the manuscript. RH, JK, and MA wrote the final version. All authors contributed to manuscript revision and read and approved the submitted version.

## Conflict of Interest

The authors declare that the research was conducted in the absence of any commercial or financial relationships that could be construed as a potential conflict of interest. The handling editor declared a past co-authorship with one of the authors JK.
