# Supplementary material for: Bumblebee Behavior on Flowers, but Not Initial Attraction, Is Altered by Short-Term Drought Stress
Source: Front Plant Sci. 2021 Jan 13;11:564802. doi: 10.3389/fpls.2020.564802 (PMC7838097; doi:10.3389/fpls.2020.564802)
Supplement: Supplementary file 1 [file Data_Sheet_1.docx]

Supplementary Material

# Supplementary Tables

**Supplementary Table 1.** Linear mixed model results testing for the effect of treatment on morphological traits. Mean (SD) are given. Significant *p* values are boldfaced.

|  | **Mean (SD)** | |  |  |
| --- | --- | --- | --- | --- |
| **Trait** | **Watered** | **Drought stressed** | ***χ^2^*** | ***p*** |
| Flower height | 47.5 (11.5) cm | 37.2 (13.1) cm | 4.03 | **0.045** |
| Display size flower | 14.6 (2.4) mm | 14.2 (2.4) mm | 0.59 | 0.442 |
| Display size inflorescences | 33.1 (8.8) mm | 32.1 (9.3) mm | 0.22 | 0.641 |
| N° flowers per plant | 10.1 (5.8) | 9.9 (7.7) | 0.02 | 0.893 |
| N° inflorescences per plant | 2.8 (1.5) | 2.5 (2.2) | 0.33 | 0.567 |
| Calyx length | 4.9 (0.5) mm | 4.9 (0.8) mm | 0.23 | 0.635 |
| Stamen length | 7.8 (0.8) mm | 7.6 (1.0) mm | 1.06 | 0.303 |
| Style length | 7.4 (0.7) mm | 7.0 (1.2) mm | 2.05 | 0.153 |
| Nectar volume per flower | 0.006 (0.012) µl | 0.003 (0.005) µl | 2.60 | 0.107 |

**Supplementary Table 2.** Scent bouquet of *S. arvensis* flowers at different temperatures and treatments. For all compounds emission rate (mean ± SE) in ng/h/flower and retention index (RI) are given. Selected compounds were found in at least four plant individuals; number of individuals is given in brackets.

|  | |  | **Mean ± SE** | | | | | |
| --- | --- | --- | --- | --- | --- | --- | --- | --- |
| **Compounds** | | **RI** | **Control 20 °C** | **Control 25 °C** | **Control 30 °C** | **Drought 20 °C** | **Drought 25 °C** | **Drought 30 °C** |
|  | Total volatiles |  | 6.75 ± 1.99 **(12)** | 8.54 ± 2.47 **(12)** | 5.80 ± 1.41 **(12)** | 9.92 ± 5.19 **(12)** | 20.91 ± 11.52 **(11)** | 5.83 ± 2.58 **(12)** |
| *Aliphatics* | |  |  |  |  |  |  |  |
|  | (*Z*)-1,5-Octadien-3-ol | 973 | 0.01 **(1)** | 0.003**(1)** | 0.1 ± 0.07 **(2)** | 0.02 **(1)** | 0.08 ± 0.06 **(3)** | 0.05 **(1)** |
| *Monoterpenes* | |  |  |  |  |  |  |  |
|  | α-Pinene | 932 | 2.19 ± 0.98 **(9)** | 1.82 ± 0.78 **(10)** | 0.78 ± 0.21 **(9)** | 0.80 ± 0.22 **(8)** | 2.14 ± 0.95 **(10)** | 0.71 ± 0.34 **(8)** |
|  | Sabinene | 972 | 0.12 ± 0.06 **(5)** | 0.24 ± 0.15 **(6)** | 0.06 ± 0.04 **(2)** | 0.03 ± 0.02 **(3)** | 0.01 **(1)** | 0.05 ± 0.04 **(2)** |
|  | β-Pinene | 977 | 0.16 ± 0.07 **(6)** | 0.22 ± 0.12 **(5)** | 0.03 ± 0.02 **(2)** | 0.06 ± 0.03 **(4)** | 0.004 **(1)** | 0.03 ± 0.02 **(2)** |
|  | β-Myrcene | 989 | 0.24 ± 0.09 **(6)** | 0.68 ± 0.38 **(5)** | 0.58 ± 0.17 **(8)** | 0.06 ± 0.03 **(4)** | 0.69 ± 0.41 **(5)** | 0.43 ± 0.29 **(4)** |
|  | Limonene | 1029 | 0.27 ± 0.11 **(6)** | 0.60 ± 0.28 **(7)** | 0.51 ± 0.26 **(4)** | 0.75 ± 0.52 **(4)** | 0.28 ± 0.14 **(6)** | 0.44 ± 0.34 **(3)** |
|  | β-Phellandrene | 1031 | 0.22 ± 0.08 **(7)** | 0.79 ± 0.38 **(7)** | 0.49 ± 0.13 **(8)** | 0.12 ± 0.06 **(5)** | 0.62 ± 0.35 **(8)** | 0.35 ± 0.22 **(4)** |
|  | (*Z*)-Ocimene | 1036 | 0.02 ± 0.21 **(1)** | 0 **(0)** | 0.03 **(1)** | 0.04 **(1)** | 0.07 ± 0.04 **(2)** | 0 **(0)** |
|  | (*E*)-β-Ocimene | 1046 | 0.40 ± 0.21 **(7)** | 1.15 ± 0.57 **(8)** | 0.48 ± 0.24 **(5)** | 0.37 ± 0.34 **(4)** | 3.96 ± 2.79 **(5)** | 0.38 ± 0.23 **(4)** |
|  | 1,3,8-p-Menthatriene | 1130 | 0 **(0)** | 0.004 **(1)** | 0.05 **(1)** | 0.05 **(1)** | 0 **(0)** | 0 **(0)** |
|  | α-Terpineol | 1196 | 0.05 ± 0.04 **(2)** | 0 **(0)** | 0.07 ± 0.05 **(2)** | 0.01 **(1)** | 0 **(0)** | 0.11 ± 0.08 **(2)** |
|  | Verbenone | 1208 | 0.16 ± 0.08 **(5)** | 0.80 ± 0.30 **(9)** | 0.47 ± 0.15 **(7)** | 0.18 ± 0.09 **(6)** | 0.75 ± 0.41 **(7)** | 0.52 ± 0.33 **(5)** |
| *Phenylpropanoids* | |  |  |  |  |  |  |  |
|  | Acetophenone | 1065 | 0.14 ± 0.07 **(4)** | 0.06 ± 0.06 **(2)** | 0.07 ± 0.04 **(3)** | 0.38 ± 0.26 **(4)** | 0.42 ± 0.40 **(2)** | 0.08 ± 0.04 **(4)** |
| *Furane* | |  |  |  |  |  |  |  |
|  | Coumaran | 1217 | 0.23 ± 0.13 **(4)** | 0.21 ± 0.14 **(3)** | 0.15 ± 0.07 **(4)** | 1.16 ± 0.94 **(4)** | 1.05 ±0.86 **(3)** | 0.10 ± 0.05 **(3)** |
| *N-containing* *compounds* | |  |  |  |  |  |  |  |
|  | Indole | 1292 | 0.19 ± 0.10 **(4)** | 0.09 ± 0.08 **(3)** | 0.13 ± 0.06 **(4)** | 0.81 ± 0.61 **(4)** | 0.60 ± 0.57 **(2)** | 0.10 ± 0.05 **(3)** |
| *Sesquiterpenes* | |  |  |  |  |  |  |  |
|  | α-Longipinene | 1352 | 0.04 **(1)** | 0.18 ± 0.12 **(3)** | 0.20 ± 0.10 **(4)** | 0.07 ± 0.05 **(2)** | 1.42 ± 0.92 **(4)** | 0.63 ± 0.38 **(4)** |
|  | Caryophyllene | 1422 | 0.03 **(1)** | 0.05 **(1)** | 0.29 ± 0.20 **(2)** | 0.09 ± 0.05 **(3)** | 0.94 ± 0.79 **(3)** | 0.75 ± 0.72 **(2)** |
| *Unknown* *compounds* | |  |  |  |  |  |  |  |
|  | Unknown3 | 1134 | 0.02 **(1)** | 0.17 ± 0.08 **(4)** | 0.03 **(1)** | 0 **(0)** | 0.20±0.12 **(3)** | 0.05 **(1)** |
|  | Unknown13 | 1145 | 0.14 ± 0.09 **(3)** | 0.06 **(1)** | 0.05 ± 0.03 **(2)** | 0.05 ± 0.04 **(2)** | 0.89 ± 2.86 **(2)** | 0.11 ± 0.06 **(3)** |
|  | Unknown6 | 1173 | 0.32 ± 0.17 **(6)** | 0.14 ± 0.13 **(2)** | 0.22 ± 0.12 **(3)** | 0.81 ± 0.50 **(4)** | 1.85±1.20 **(4)** | 0.18 ± 0.09 **(3)** |
|  | Unknown14 | 1392 | 0.05 ± 0.05 **(2)** | 0.01 **(1)** | 0 **(0)** | 0.02 **(1)** | 0.03 **(1)** | 0.02 **(1)** |
|  | Unknown5 | 1466 | 0.02 **(1)** | 0.07 ± 0.04 **(3)** | 0.07 ± 0.05 **(2)** | 0 **(0)** | 0.09 **(1)** | 0.05 **(1)** |
|  | Unknown4 | 2317 | 1.64 ± 0.69 **(8)** | 1.13 ± 0.47 **(9)** | 0.88 ± 0.32 **(6)** | 4.04 ± 2.56 **(9)** | 4.73 ± 3.26 **(6)** | 0.66 ± 0.28 **(5)** |
|  | Unknown11 | 2434 | 0.06 **(1)** | 0.04 ± 0.04 **(2)** | 0.06 **(1)** | 0 **(0)** | 0.08 ± 0.07 **(2)** | 0.01 **(1)** |

|  | | Floral traits | | | | | | | | | | | | | |
| --- | --- | --- | --- | --- | --- | --- | --- | --- | --- | --- | --- | --- | --- | --- | --- |
|  | | Display size inflorescences | | Display size flower | | N° inflorescences per plant | | N° flowers per plant | | Flower height | | Nectar volume per flower | | Scent emission | |
|  | | ***χ^2^*** | ***p*** | ***χ^2^*** | ***p*** | ***χ^2^*** | ***p*** | ***χ^2^*** | ***p*** | ***χ^2^*** | ***p*** | ***χ^2^*** | ***p*** | ***χ^2^*** | ***p*** |
| Time to first visit | |  | | | | | | | | | | | | | |
|  | trait | 4.44 | **0.035** | 3.39 | 0.066 | 1.31 | 0.252 | 0.4 | 0.528 | 1.21 | 0.272 | 2.74 | 0.098 | 3.20 | 0.073 |
|  | treatment | 1.28 | 0.259 | 1.00 | 0.317 | 1.11 | 0.292 | 1.25 | 0.263 | 2.07 | 0.150 | 1.03 | 0.311 | 2.28 | 0.131 |
|  | trait x treat | 0.03 | 0.863 | 4.61 | **0.032** | 0.14 | 0.707 | 0.33 | 0.563 | 0.21 | 0.647 | 0.02 | 0.887 | 0.21 | 0.647 |
| N° visits per plant | |  | | | | | | | | | | | | | |
|  | trait | 23.23 | **<0.001** | 1.30 | 0.254 | 23.18 | <**0.001** | 26.76 | **<0.001** | 1.36 | 0.243 | 7.54 | **0.006** | 22.61 | **<0.001** |
|  | treatment | 9.08 | **0.003** | 7.41 | **0.006** | 6.07 | **0.014** | 6.06 | **0.014** | 4.43 | **0.035** | 10.36 | **0.001** | 15.47 | **<0.001** |
|  | trait x treat | 0.8 | 0.372 | 7.63 | **0.006** | 0.001 | 0.981 | 0.49 | 0.486 | 0.12 | 0.725 | 0.13 | 0.714 | 6.01 | **0.014** |
| Visitation rate | |  | | | | | | | | | | | | | |
|  | trait | 6.08 | **0.014** | 5.48 | **0.019** | 0.66 | 0.416 | 0.31 | 0.575 | 0.03 | 0.859 | 2.28 | 0.131 | 3.67 | 0.055 |
|  | treatment | 9.85 | **0.002** | 8.21 | **0.004** | 9.31 | **0.002** | 9.01 | **0.003** | 7.64 | **0.006** | 7.61 | **0.006** | 9.66 | **0.002** |
|  | trait x treat | 0.05 | 0.813 | 0.15 | 0.702 | 2.08 | 0.149 | 0.56 | 0.455 | 2.67 | 0.102 | 1.37 | 0.242 | 1.31 | 0.252 |
| Rel. landing duration | |  | | | | | | | | | | | | | |
|  | trait | 1.20 | 0.273 | 0.17 | 0.677 | 2.74 | 0.098 | 2.81 | 0.094 | 0.50 | 0.479 | 1.25 | 0.264 | 0.1 | 0.754 |
|  | treatment | 4.36 | **0.037** | 4.34 | **0.037** | 4.68 | **0.031** | 4.54 | **0.033** | 2.94 | 0.086 | 4.03 | **0.045** | 3.73 | 0.053 |
|  | trait x treat | 0.61 | 0.433 | 0.05 | 0.827 | 2.29 | 0.13 | 0.90 | 0.342 | 2.4 | 0.121 | 0.38 | 0.537 | 0.64 | 0.422 |

**Supplementary Table 3.** Linear mixed model results testing the effect of treatment and floral traits on bumblebee behavior. Significant *p* values are boldfaced; underlined numbers indicate *p* < 0.1.

# Supplementary Figures


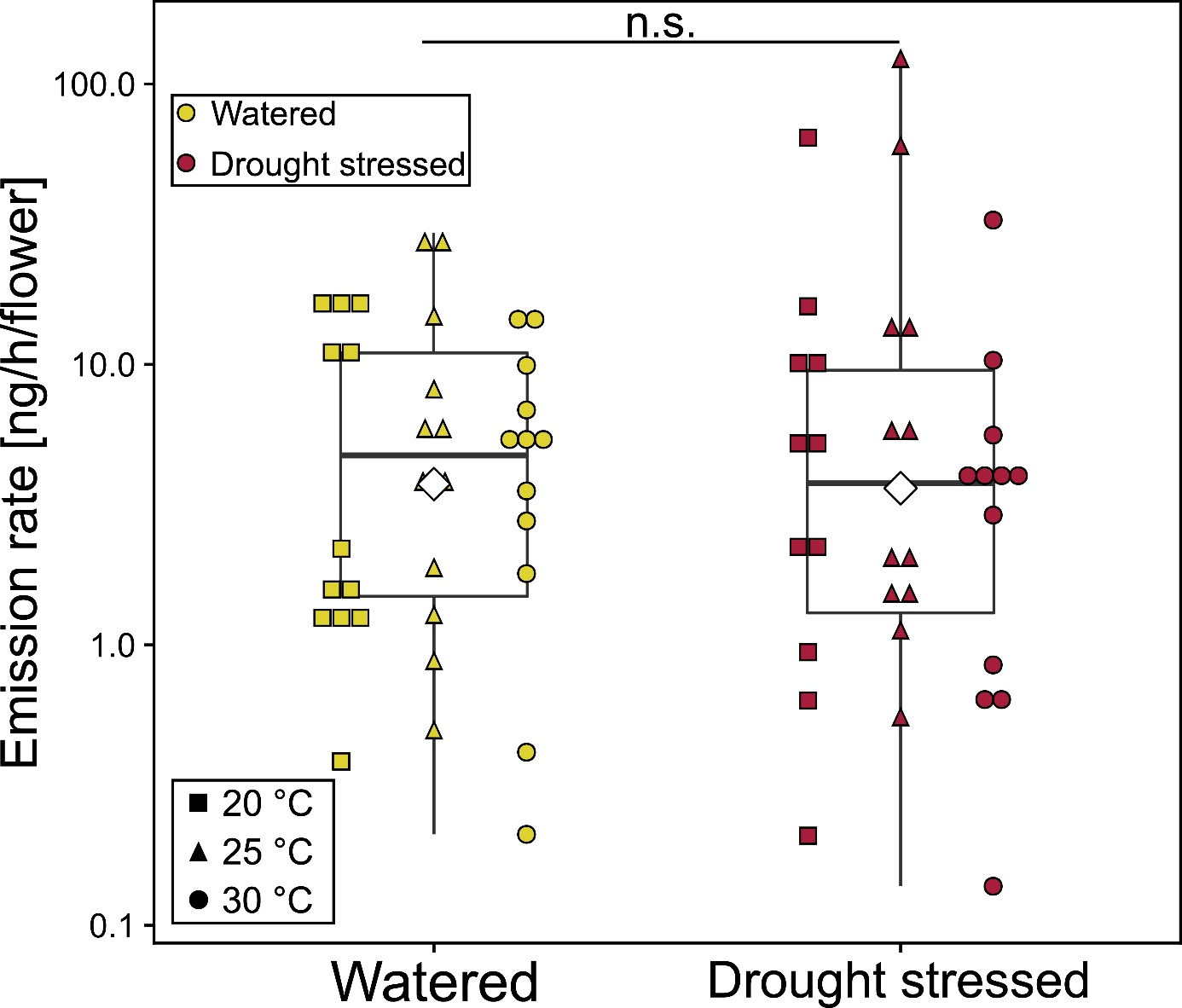


**Supplementary Figure 1.** Total scent emission rate [ng/h/flower] of watered and drought stress plants at different temperatures. Y-axis in logarithmic scale. Each colored dot represents one plant individual. Watered: N = 36 (mean (SD) 7.06 (7.32) ng h^-1^ per flower); drought stressed: N = 35 (mean (SD) 11.97 (24.5) ng h^-1^ per flower). Different symbols show emission rate at different temperature. Boxplots show median, interquartile range and minimum/maximum range. White diamonds show mean value. n.s., non-significant.


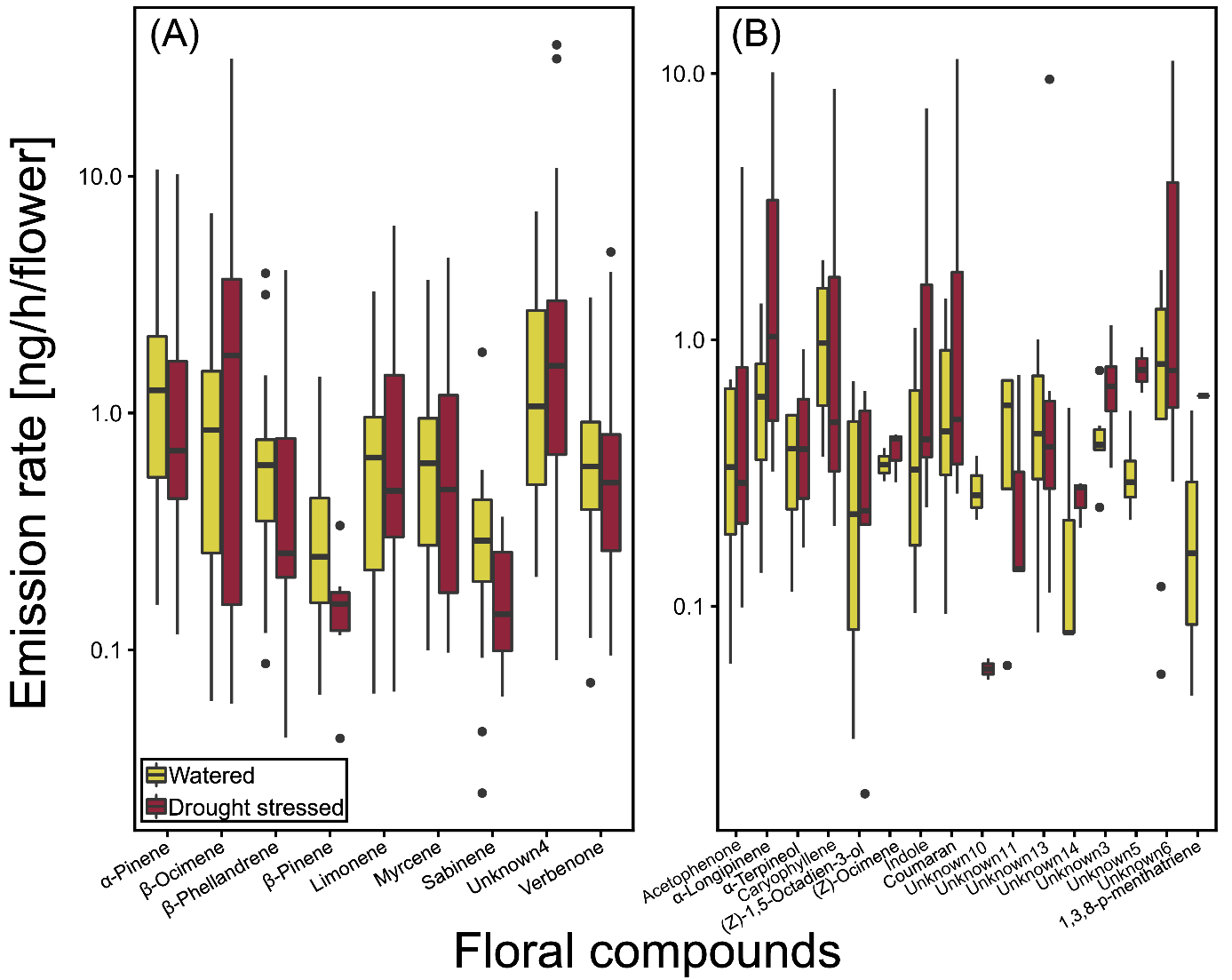


**Supplementary Figure 2.** Emission rate [ng/h/flower] of the **(A)** most common floral compounds and **(B)** less common floral compounds in scent bouquet of watered and drought stressed plants. Y-axis in logarithmic scale. Boxplots show median, interquartile range and minimum/maximum range, black dots show outliers.


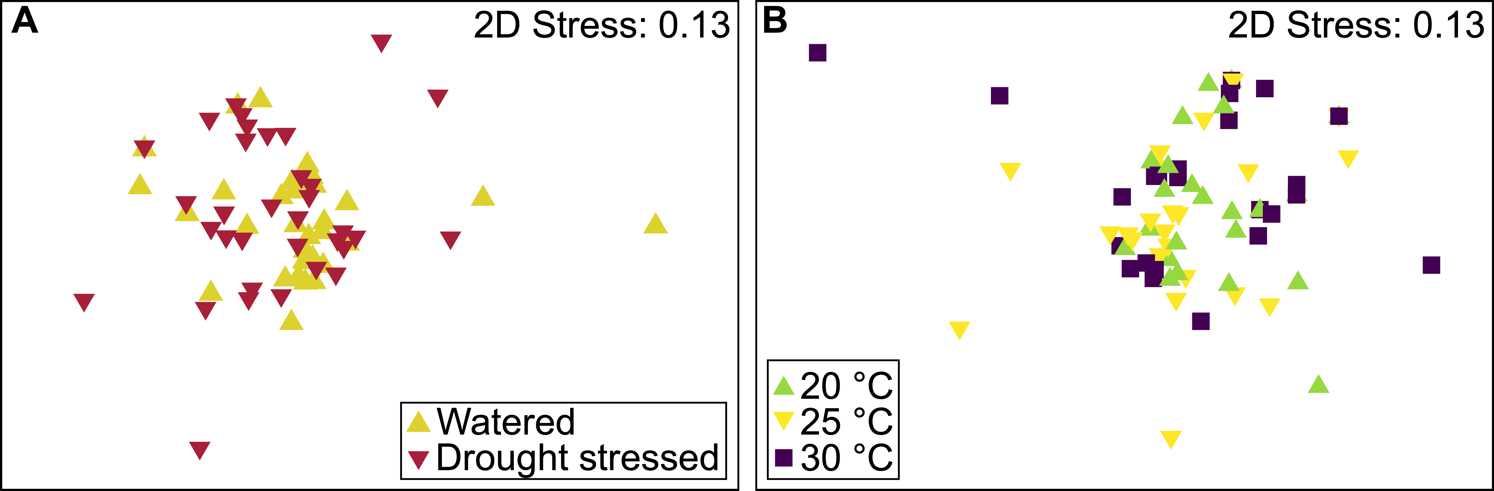


**Supplementary Figure 3.** Scent bouquet of plant individuals of both treatments (A) and at different temperatures (B); MDS, Bray-Curtis similarity measures, 2D stress: 0.13, PERMANOVA (temperature: Pseudo-*F*_2,65_ = 1.06, *p* = 0.385; treatment: Pseudo-*F*_1,65_ = 0.81, *p* = 0557; treatment × temperature: Pseudo-*F*_2,65_ = 1.07, *p* = 0.363).
